# Cervical cancer screening preferences by sexual orientation among persons assigned female at birth

**DOI:** 10.1186/s12905-026-04678-7

**Published:** 2026-07-17

**Authors:** Wei Yi Kong, Jennifer L. St. Sauver, Robert M. Jacobson, Kathy L. MacLaughlin, Lila J. Finney Rutten

**Affiliations:** 1 Division of Epidemiology, Department of Quantitative Health Sciences, Mayo Clinic, Rochester, MN USA; 2 Division of Community Pediatric and Adolescent Medicine, Department of Pediatric and Adolescent Medicine, Mayo Clinic, Rochester, MN USA; 3 Division of Pediatric Infectious Diseases, Department of Pediatric and Adolescent Medicine, Mayo Clinic, Rochester, MN USA; 4 Department of Family Medicine, Mayo Clinic, Rochester, MN USA; 5 Division of Community Internal Medicine, Geriatrics, and Palliative Care, Department of Medicine, Mayo Clinic, Rochester, MN USA; 6 Jefferson College of Population Health, Thomas Jefferson University, Philadelphia, PA USA

**Keywords:** Early detection of cancer, Uterine cervical neoplasms, Sexual and gender minorities, Sexual behavior, Self-testing

## Abstract

**Background:**

Disparities in up-to-date cervical cancer screening rates persist among women, including those identifying as lesbian, gay, bisexual or other sexual orientation (LGB+), highlighting the need for more inclusive and targeted screening interventions. With human papillomavirus at-home self-testing recently approved, we studied cervical cancer screening preferences by sexual orientation among persons assigned female at birth.

**Methods:**

We examined cervical cancer screening preferences among a nationally representative sample of persons assigned female at birth aged 25–65 years in the 2024 Health Information National Trends Survey who did not self-report a history of cervical cancer. We assessed variation in preference for at-home self-testing or clinician-delivered cervical cancer screening by sexual orientation using weighted Rao-Scott chi-square tests and multivariate multinomial regression. We also examined reasons for preferring at-home self-testing.

**Results:**

The analytical sample included 2,168 respondents. Most self-identified as heterosexual (88.43%) and preferred cervical cancer screening by clinicians (61.45%). Cervical cancer screening preferences differed by sexual orientation (*p* = 0.02). LGB+ versus heterosexual respondents more frequently preferred at-home self-testing (30.97% versus 19.53%), whereas heterosexual respondents more frequently preferred testing by clinician than their LGB+ counterparts (62.81% versus 50.38%). The odds of preferring at-home self-testing over clinician-delivered screening were higher among LGB+ versus heterosexual respondents (adjusted odds ratio = 2.04, 95% confidence interval = 1.17˗3.53), after adjusting for age, household annual income, marital status, health care utilization, race/ethnicity, and health insurance. The most frequently endorsed reason for preferring at-home self-testing was privacy (66.25%). Preferring at-home self-testing due to not taking time off work (35.22%), saving transportation cost (34.24%), and avoiding embarrassment (39.41%) were endorsed less frequently. Reasons for preferring at-home self-testing did not differ by sexual orientation.

**Conclusion:**

LGB+ respondents more frequently preferred at-home self-testing compared to heterosexual respondents. These findings highlight the potential utility of at-home self-testing in addressing screening preferences and barriers among LGB+ persons.

**Supplementary Information:**

The online version contains supplementary material available at https://doi.org/10.1186/s12905-026-04678-7.

## Background

Cervical cancer is highly preventable with routine screening for human papillomavirus (HPV) and precancers, in addition to HPV vaccination. However, up-to-date cervical cancer screening among females aged 21–65 years in the United States (US) was 75.8% in 2023 [1], below the national public health goals [2]. Furthermore, substantial disparities in screening adherence persist, with lower up-to-date screening rates observed among specific populations, including females of diverse sexual orientations, particularly when intersecting with racial and ethnic identities [3–6]. Most recent estimates indicate that up-to-date screening prevalence was 8.28% lower among females identified as lesbian, gay, or bisexual versus those who identified as heterosexual [4]. Additionally, the odds of up-to-date screening was 63% lower among lesbian, gay, or bisexual non-Hispanic Black females, compared to heterosexual non-Hispanic White females [4]. The lack of awareness regarding needing to screen was the most commonly cited reason among a national sample of females, including those who identified as lesbian, gay, bisexual, or other sexual orientation (LGB+), for not being up-to-date on their cervical cancer screening [7]. Experiences of less culturally competent primary care among LGB+ females may further hinder cervical cancer screening uptake [8–10].

In May 2024, HPV self-collection for cervical cancer screening in healthcare setting was approved with specific lab platforms and partnered devices [11]. More recently, two at-home HPV self-testing kits received approval or clearance as alternative cervical cancer screening options [12, 13]. Intervention studies and cross-sectional surveys have consistently suggested the potential for at-home self-testing to boost screening rates [14–20]. At-home self-testing may represent an opportunity to address disparities in screening uptake by sexual orientation and intersecting racial and ethnic identities. However, research assessing the acceptability of at-home self-testing among LGB+ females remains limited and has yielded mixed findings [21–23]. Reasons for preferring at-home self-testing among LGB+ females are also not explored in these existing studies [21–23]. We aimed to provide further insights on cervical cancer screening preferences by sexual orientation and to examine the potential role of self-testing in closing screening gaps among LGB+ females.

## Methods

### Study design

We constructed an analytical sample from the public-use Health Information National Trends Survey (HINTS 7) dataset, which was collected in March-September 2024. HINTS 7 was targeted at a nationally representative sample of noninstitutionalized civilian US adults via a two-stage stratified sampling design and administered with paper and web-based options [24]. HINTS 7 received 7,278 eligible non-duplicate responses (27.3% response rate) with fully or partially completed questionnaires [24].

We narrowed our analytical sample to participants who: 1) indicated that they were assigned female at birth on their birth certificate; 2) were aged 25–65 years, consistent with American Cancer Society guidelines recommending primary HPV-based screening in this age group [25, 26]; and 3) did not report a history of cervical cancer. Therefore, we excluded participants who were not assigned female at birth, were outside the prespecified age range, or had a self-reported cervical cancer diagnosis. HINTS data collection received approval from the US Office of Management and Budget and underwent review by the National Institutes of Health Office of Human Subjects Research Protection. As the dataset is deidentified and publicly available, the Mayo Clinic Institutional Review Board determined that our analysis of this data set was exempt from IRB review.

### Survey measures

Participants reported their sexual orientation, age, race/ethnicity, household income, health insurance, education, employment status, marital status, health care utilization, and trust in the health care system [27]. For sexual orientation, participants were asked: “Do you think of yourself as…” (1=“Lesbian or gay,” 2=“Straight, that is, not gay or lesbian,” 3=“Bisexual,” 4=“I use a different term,” 5=“Don’t know”). Open-ended responses to “I use a different term,” such as asexual, pansexual, and queer, were recoded into “something else.” To assess screening preferences among LGB+ respondents, we collapsed all response categories, excluding “straight.” We collapsed “Don’t know” responses in the LGB+ subgroup to include participants who may be questioning their sexual orientation. We also collapsed some response categories in household income, education, employment status, marital status, health care utilization, and trust in the health care system due to small cell sizes.

HINTS 7 was fielded before the approval of an at-home self-testing kit [12]. Participants were given a description of a hypothetical at-home self-testing option: “Cervical cancer screening helps to prevent and detect cervical cancer in people who have no symptoms. In the future, it may be possible for people to collect their own sample for the cervical cancer screening test at home using a mailed test kit. This might involve using a swab (like a long cotton bud) to take a sample from the vagina or by collecting a sample of urine.”

Participants were then asked if they would prefer cervical cancer screening by a clinician, at-home self-testing, unsure, or not applicable. We excluded those who selected “Not applicable – I do not need cervical cancer screening” as all participants had received this survey question, including those assigned male at birth and those who may have had a history of hysterectomy. Participants who preferred at-home self-testing or were unsure were then invited to indicate all their reasons for considering self-testing from a list of options.

### Statistical analysis

We conducted Rao-Scott chi-square tests to describe participants’ sociodemographic and psychosocial characteristics, cervical cancer screening preferences, and reasons for considering at-home self-testing. Next, we ran multivariate multinomial regression to examine screening preferences of LGB+ versus heterosexual respondents, adjusting for all sociodemographic and psychosocial characteristics that differed significantly by sexual orientation. We additionally adjusted for race/ethnicity and health insurance, which were previously found to be associated with cervical cancer screening [3, 4, 22]. Preference for clinician-delivered screening was designated as the reference outcome, and the model estimated the odds of preferring at-home self-testing versus clinician screening and being unsure versus clinician screening among LGB+ versus heterosexual respondents. We also explored screening preferences by intersecting identities of sexual orientation and race/ethnicity.

Then, among participants who preferred at-home self-testing, we conducted stratified multivariate logistic regression to assess if each of the pre-specified reasons for preferring at-home self-testing varied by sexual orientation, adjusting for the same sociodemographic and psychosocial characteristics. We omitted reasons for preferring at-home self-testing with fewer than 25 responses. We also did not present our analysis of reasons for preferring at-home self-testing among participants who were unsure about their cervical cancer screening preference due to small cell sizes. The small sample of LGB+ respondents precluded our ability to further explore their reasons for preferring at-home self-testing by their race/ethnicity.

We used a *p*-value of 0.05 to determine statistical significance in our analyses conducted in SAS version 9.4 (SAS Institute, Cary, NC). Given the complex sample survey design, we included a final sample weight and 50 replicate weights using the delete-one jackknife method to examine variations in screening preferences and reasons. Listwise deletion was used to handle missing data (≤ 2.31% across study variables).

## Results

Table 1 presents the characteristics of the HINTS 7 sample of respondents aged 25–65 years (*N* = 2,168), of whom 11.57% self-identified as LGB+. LGB+ participants were significantly younger (21.82% aged 25–29 years versus 8.84% among heterosexual respondents, *p* < 0.0001), were more likely to report lower household income (*p* = 0.005), were less likely to be married or living with a partner (37.59% versus 64.85%, *p* < 0.0001) and had differences in health care utilization (*p* = 0.03). No significant differences were observed by sexual orientation for race/ethnicity, health insurance, education, employment status, or trust in the health care system.

**Table 1:** Health Information National Trends Survey 7 participant characteristics aged 25–65 years (*N* = 2168)

Characteristic	Overall (unweighted)		Sexual orientation (weighted)				
			Heterosexual		Lesbian, gay, bisexual, or other sexual orientation^a^		
	*n*	(%)	*n*	(%)	*n*	(%)	ꭕ^2^ * p*-value
Overall sample	2168	(100)	1873	(88.43)	245	(11.57)	
Age, in years							**< 0.0001**
25–29	208	(9.59)	151	(8.84)	53	(21.82)	
30–49	1024	(47.23)	862	(49.78)	140	(64.83)	
50–65	936	(43.17)	860	(41.38)	52	(13.35)	
Race/ethnicity							0.44
Non-Hispanic White	1055	(49.62)	913	(62.24)	128	(59.85)	
Non-Hispanic Black	361	(16.98)	328	(12.05)	29	(8.15)	
Hispanic	515	(24.22)	432	(17.51)	59	(20.51)	
Non-Hispanic Other^b^	195	(9.17)	165	(8.20)	27	(11.49)	
Sexual orientation and Race/ethnicity							
Heterosexual non-Hispanic White	913	(43.87)	–		–		
Heterosexual non-Hispanic Black	328	(15.76)	–		–		
Heterosexual Hispanic	432	(20.76)	–		–		
Heterosexual non-Hispanic Other	165	(7.93)	–		–		
LGB + non-Hispanic White	128	(6.15)	–		–		
LGB + non-Hispanic Black	29	(1.39)	–		–		
LGB+ Hispanic	59	(2.84)	–		–		
LGB + non-Hispanic Other	27	(1.30)	–		–		
Household annual income							**0.005**
<$20,000	342	(15.78)	268	(12.52)	53	(25.33)	
$20,000 to <$50,000^c^	455	(21.00)	402	(21.49)	45	(17.81)	
$50,000 to <$75,000	359	(16.57)	312	(14.23)	41	(20.17)	
$75,000 to <$100,000	292	(13.47)	256	(14.22)	33	(12.58)	
≥$100,000	719	(33.18)	634	(37.55)	73	(24.11)	
Health insurance coverage							0.33
Yes	1910	(88.43)	1661	(92.38)	211	(89.85)	
No	250	(11.57)	205	(7.62)	34	(10.15)	
Education level completed							0.50
High school or less^c^	411	(19.05)	338	(26.41)	49	(26.03)	
Some college	547	(25.35)	481	(35.11)	57	(29.22)	
Bachelor’s degree	712	(32.99)	628	(21.87)	77	(24.81)	
Post-baccalaureate degree	488	(22.61)	419	(16.61)	61	(19.95)	
Occupation status^d^							0.67
Employed, homemaker, or student^c^	1520	(71.26)	1328	(71.87)	164	(69.21)	
Unemployed,^e^ retired, or disabled^c^	352	(16.50)	303	(15.88)	36	(15.36)	
Multiple statuses	261	(12.24)	216	(12.26)	39	(15.43)	
Marital status							**< 0.0001**
Married or living with a partner^c^	1143	(52.84)	1025	(64.85)	99	(37.59)	
Divorced, widowed, or separated^c^	475	(21.96)	417	(8.38)	47	(8.22)	
Single, never married	545	(25.20)	429	(26.77)	98	(54.19)	
Health care visits in the past 12 months							**0.03**
None	220	(10.16)	183	(8.69)	31	(13.87)	
1–2 times^c^	662	(30.58)	579	(30.31)	66	(21.25)	
3 times	340	(15.70)	307	(17.23)	29	(11.62)	
4 times	308	(14.23)	257	(12.72)	42	(21.88)	
5–9 times	388	(17.92)	344	(20.47)	35	(14.23)	
10 + times	247	(11.41)	200	(10.58)	42	(17.14)	
Trust in the health care system							0.89
Not at all/A little^c^	422	(19.52)	346	(21.96)	65	(24.12)	
Some	1076	(49.77)	941	(47.81)	111	(46.60)	
A lot	664	(30.71)	580	(30.23)	69	(29.28)	

Boldface indicates statistical significance at *p* ≤ 0.05

^a^Includes bisexual, gay, lesbian, “something else,” and “don’t know”

^b^Includes Alaska Native, American Indian, Asian, Native Hawaiian, other Pacific Islander, and multiple races

^c^Categories were collapsed due to small cell sizes with less than 25 responses

^d^Excludes “Other Occupation Only” (*n* = 26)

^e^Includes unemployed for < 1 year and unemployed for ≥ 1 year

### Cervical cancer screening preferences

Among our analytical sample, 61.45% preferred cervical cancer screening by clinicians, 20.46% preferred at-home self-testing, and 18.10% were unsure (Table 2). Screening preferences differed by sexual orientation (*p* = 0.02). Specifically, LGB+ versus heterosexual respondents more frequently preferred at-home self-testing (30.97% versus 19.53%), while heterosexual respondents more frequently preferred testing by clinician than their LGB+ counterparts (62.81% versus 50.38%). Controlling for age, household income, marital status, health care utilization, race/ethnicity, and health insurance, LGB+ versus heterosexual respondents were more likely to prefer at-home self-testing (adjusted odds ratio [aOR] = 2.04, 95% confidence interval [CI] = 1.17–3.53).

**Table 2:** Cervical cancer screening preference and reasons for preferring HPV self-testing (*N* = 2168)

	Overall sample (weighted)		Sexual orientation (weighted)							
			Heterosexual			Lesbian, gay, bisexual, or other sexual orientation				
	*n*	(%)	*n*		(%)	*n*		(%)	ꭕ^2^ * p*-value	Adjusted OR^c^ (95% CI) for LGB+ versus heterosexual
Cervical cancers creening preference									**0.02**	
Clinician	1325	(61.45)	1178		(62.81)	115		(50.38)		Reference
At-home self-testing	440	(20.46)	361		(19.53)	72		(30.97)		**2.04 (1.17**-**3.53)**
Unsure	403	(18.10)	334		(17.66)	58		(18.65)		1.31 (0.73-2.35)
Reasons for preferring at-home self-testing (*n* = 440)^a, b^										
Not take time off work	173	(35.22)	147		(35.10)	25		(38.88)	0.69	1.30 (0.47-3.60)
Save transportation cost	140	(34.24)	110		(31.54)	26		(49.09)	0.10	1.50 (0.34-6.60)
Privacy	285	(66.25)	235		(66.74)	45		(64.14)	0.79	0.66 (0.26-1.67)
Avoid embarrassment	143	(39.41)	111		(36.52)	29		(54.71)	0.09	1.43 (0.57-3.62)

*CI*  Confidence interval, *OR*  Odds ratio, *LGB* +  Lesbian, gay, bisexual or other sexual orientation

Boldface indicates statistical significance at *p *≤0.05

^a^Omitted results on “Live far from clinicians” and “Other reasons” due to small sample sizes with less than 25 responses

^b^Values do not add up to 100% due to multiple responses

^c^Adjusted for age, household annual income, marital status, health care utilization, race/ethnicity, and health insurance

Screening preferences differed among some respondents by their intersecting sexual orientation and racial/ethnic identities (Supplemental Table S1). Compared with heterosexual non-Hispanic White respondents, the likelihood of preferring at-home self-testing over clinician-performed screening was higher among LGB + non-Hispanic White respondents (aOR = 2.25, 95% CI = 1.11–4.55) but lower among heterosexual Hispanic respondents (aOR = 0.42, 95% CI = 0.21–0.83). No significant differences in preference between at-home self-testing and clinician-performed screening were observed for the other subgroups.

### Reasons for preferring at-home self-testing

Among those who indicated a preference for at-home self-testing (*n* = 440), the most frequently endorsed reason for preferring at-home self-testing was privacy (66.25%) (Table 2). Not taking time off work (35.22%), saving transportation cost (34.24%), and avoiding embarrassment (39.41%) were endorsed less frequently. Reasons for preferring at-home self-testing did not differ significantly by sexual orientation among those who indicated a preference for at-home self-testing (range of *p*-values: 0.09–0.79).

## Discussion

In our analysis of HINTS 7, LGB+ respondents were over two times more likely to prefer at-home HPV self-testing compared to heterosexual respondents. This association remained even after adjusting for other sociodemographic characteristics (age, household annual income, marital status, health care utilization, race/ethnicity, and health insurance). Our study finding contrasts with the observed nonsignificant difference in screening preference by sexual orientation in one analysis of HINTS 7 [22] but is consistent with an analysis of another national survey data in that LGB+ respondents more frequently prefer at-home self-testing [23]. We speculate that the inconsistency across all study findings may be in part due to differences in the study samples by age and operationalization of sexual orientation. Nevertheless, our study adds to the extant literature by comparing reasons for preferring at-home self-testing between LGB + and heterosexual respondents.

Reasons for considering at-home self-testing were generally similar between heterosexual and LGB+ respondents who indicated a preference for at-home self-testing, with privacy cited most frequently by all participants. Although LGB+ participants tended to report a greater preference for less embarrassment and lower transportation costs, none of these differences reached statistical significance after adjustment. With other research indicating that LGB+ individuals were similarly lacking awareness of the necessity to screen [7] but experience less culturally competent primary care than heterosexual individuals [8–10], our findings suggest that at-home HPV self-testing may help address important screening barriers among LGB+ respondents and could represent one strategy to reduce disparities in cervical cancer screening by sexual orientation. Engaging in culturally sensitive and inclusive efforts of offering LGB+ respondents the option of at-home self-testing could strengthen their awareness and access to cervical cancer screening.

Our exploratory analysis suggested differences in screening preferences among some respondents by their intersecting racial/ethnic identities. We observed that, relative to heterosexual non-Hispanic White respondents, at-home self-testing preference was significantly higher among LGB + non-Hispanic White respondents but not in LGB+ respondents who identified as Black, Hispanic, or non-Hispanic Other race/ethnicities. In addition, heterosexual Hispanic respondents were less likely to report at-home self-testing as their screening preference compared to heterosexual non-Hispanic White respondents. This significant difference in screening preference between heterosexual Hispanic and non-Hispanic White respondents contrasts the nonsignificant finding observed in another study [23] and merits further study to better understand facilitators and barriers to adopting at-home self-testing among those underscreened for cervical cancer. Although our analytical sample size precluded our ability to analyze LGB+ respondents’ stronger preference for screening by a clinician, our study identifies populations who could benefit from greater access to different screening options.

### Limitations

Our findings provide a snapshot of screening preferences among a nationally representative sample of respondents who were age-eligible for HPV self-testing for primary cervical cancer screening. A limitation of our study is the fielding of HINTS 7 before at-home self-testing was approved, which likely attenuated participants’ comparative evaluation between at-home self-testing and clinician-performed tests. Future studies should assess the adoption of different screening options and examine variations by sociodemographic characteristics to inform targeted interventions. The cross-sectional design of HINTS also precluded causal inferences. The small analytical sample of LGB+ respondents may have biased our model estimates, and our inclusion of survey participants who may be questioning their sexual orientation likely also affected the representativeness of our LGB+ sample.

Furthermore, all measures were subjected to potential self-reporting bias. Key constructs, including sexual orientation and reasons for screening preferences, were assessed using single-item measures, limiting the depth and nuance of our findings. Our ability to understand screening preferences more comprehensively was also limited by the lack of survey measures on reasons for preferring clinician-delivered screening. Other potential mechanisms, such as attitudes toward at-home HPV self-testing, up-to-date screening and HPV vaccination statuses, and related health conditions, were not measured in HINTS 7 but could be explored to probe the association between sexual orientation and at-home self-testing preference. Despite noted limitations, this study provides additional insights into screening preferences among LGB+ respondents and highlights opportunities to improve the inclusivity and responsiveness of screening approaches.

## Conclusion

This study provides further insights into cervical cancer screening preferences among US respondents aged 25–65 years and highlights important differences by sexual orientation. LGB+ participants were more likely than heterosexual participants to prefer at-home HPV self-testing over clinician-delivered screening, underscoring the potential of at-home self-testing in addressing screening preferences and barriers in this population. Expanding access to at-home HPV self-testing may support more equitable and person-centered cervical cancer screening. Future research should explore strategies to support informed choice and screening uptake, particularly among sexual minority populations. Future research should also examine cervical cancer screening preferences among other populations eligible for screening, including transgender and nonbinary individuals.

## Supplementary Information

Supplementary Material 1.

## Data Availability

The data analyzed in this study are publicly available from the Health Information National Trends Survey sponsored by the National Cancer Institute.

## Abbreviations

aOR: Adjusted odds ratio
CI: Confidence interval
HINTS: Health Information National Trends Survey
HPV: Human papillomavirus
LGB+: Lesbian, gay, bisexual or other sexual orientation
US: United States
