# Supplementary material for: Cervical cancer screening preferences by sexual orientation among persons assigned female at birth
Source: BMC Womens Health. 2026 Jul 17;26:514. doi: 10.1186/s12905-026-04678-7 (PMC13660203; doi:10.1186/s12905-026-04678-7)
Supplement: Supplementary file 1 [file 12905_2026_4678_MOESM1_ESM.docx]

**Supplemental Table S1.** Adjusted odds and 95% confidence interval of cervical cancer screening preference (*n*=2081^a^)

|  | Cervical cancer screening preference | | |
| --- | --- | --- | --- |
|  | Clinician | At-home self-testing | Unsure |
| **Sexual orientation and Race/ethnicity**  (Reference: Heterosexual non-Hispanic White) |  |  |  |
| Heterosexual non-Hispanic Black (*n*=328) | Reference | 0.56 (0.26, 1.22) | **0.49 (0.27, 0.89)** |
| Heterosexual Hispanic (*n*=432) | Reference | **0.42 (0.21, 0.83)** | 0.63 (0.38, 1.02) |
| Heterosexual non-Hispanic Other (*n*=165) | Reference | 0.63 (0.29, 1.36) | 0.62 (0.30, 1.28) |
| LGB+ non-Hispanic White (*n*=128) | Reference | **2.25 (1.11, 4.55)** | 1.38 (0.66, 2.89) |
| LGB+ non-Hispanic Black (*n*=29) | Reference | 0.17 (0.01, 4.88) | 1.17 (0.35, 3.88) |
| LGB+ Hispanic (*n*=59) | Reference | 1.25 (0.45, 3.49) | 0.58 (0.15, 2.26) |
| LGB+ non-Hispanic Other (*n*=27) | Reference | 0.60 (0.05, 7.55) | 0.53 (0.10, 2.71) |

*Note*. Boldface indicates statistical significance at *p*≤0.05.

*Note*. Models adjusted for age, household annual income, marital status, health care utilization, and health insurance.

^a^Missing data (*n*=87).
